# Dynamic Patterns and Modeling of Early COVID-19 Transmission by Dynamic Mode Decomposition

**DOI:** 10.5888/pcd20.230089

**Published:** 2023-10-26

**Authors:** Dehong Fang, Lei Guo, M. Courtney Hughes, Jifu Tan

**Affiliations:** 1 Department of Mechanical Engineering, Northern Illinois University, DeKalb, Illinois; 2 School of Interdisciplinary Health Professions, Northern Illinois University, DeKalb, Illinois; 3 School of Health Studies, Northern Illinois University, DeKalb, Illinois

## Abstract

**Introduction:**

Understanding the transmission patterns and dynamics of COVID-19 is critical to effective monitoring, intervention, and control for future pandemics. The aim of this study was to investigate the spatial and temporal characteristics of COVID-19 transmission during the early stage of the outbreak in the US, with the goal of informing future responses to similar outbreaks.

**Methods:**

We used dynamic mode decomposition (DMD) and national data on COVID-19 cases (April 6, 2020–October 9, 2020) to model the spread of COVID-19 in the US as a dynamic system. DMD can decompose the complex evolution of disease cases into linear combinations of simple spatial patterns or structures (modes) with time-dependent mode amplitudes (coefficients). The modes reveal the hidden dynamic behaviors of the data. We identified geographic patterns of COVID-19 spread and quantified time-dependent changes in COVID-19 cases during the study period.

**Results:**

The magnitude analysis from the dominant mode in DMD showed that California, Louisiana, Kansas, Georgia, and Texas had higher numbers of COVID-19 cases than other areas during the study period. States such as Arizona, Florida, Georgia, Massachusetts, New York, and Texas showed simultaneous increases in the number of COVID-19 cases, consistent with data from the Centers for Disease Control and Prevention.

**Conclusion:**

Results from DMD analysis indicate that certain areas in the US shared similar trends and similar spatiotemporal transmission patterns of COVID-19. These results provide valuable insights into the spread of COVID-19 and can inform policy makers and public health authorities in designing and implementing mitigation interventions.

**Summary**

**What is already known on this topic?**

People who have COVID-19 but are asymptomatic can transmit the SARS-CoV-2 virus, making it difficult to accurately model the dynamic spread of the infection.

**What is added by this report?**

We used dynamic mode decomposition to show that certain areas in the US shared similar trends and similar spatiotemporal transmission patterns of COVID-19.

**What are the implications for public health practice?**

Our findings can contribute to a better understanding of the characteristics of early COVID-19 transmission and provide decision makers with timely and accurate information to respond to the pandemic and future public health emergencies.

## Introduction

COVID-19 has caused millions of deaths and is a major public health burden worldwide. The rapid increase in COVID-19 cases can be attributed to various factors, such as the distinctive spike protein of SARS-CoV-2, the virus that causes COVID-19; the virus’ exponential growth rate, high reproduction number (R0), and high mutation rate; and poorly ventilated indoor settings (1-4). In addition, asymptomatic people may transmit COVID-19, making it difficult to accurately model the dynamic spread of the disease (5). Because of the magnitude and severity of outcomes associated with COVID-19, investigation of the coherent spatiotemporal dynamics of COVID-19 transmission is crucial (6,7).

Dynamic mode decomposition (DMD) is an equation-free method originally developed in the field of fluid dynamics to investigate coherent spatiotemporal modes. DMD can efficiently reveal the hidden structures of spatiotemporal dynamics from existing data without the requirement of previous assumptions of the studied dataset (8,9). It is a top-down data-driven model that discovers the eigenvectors and eigenvalues of a mapping matrix that relates 2 different snapshots of given data. DMD has limited applicability for strong nonlinear problems (eg, public health interventions for vaccine coverage and herd immunity that may require nonlinear modeling and feedback mechanisms) and long-range predictions (eg, prediction of obesity prevalence in a city for the next 20 years with data only from the most recent year); however, this limited applicability does not affect short-range predictions or studies on the cumulative number of disease cases. Before the COVID-19 pandemic, DMD had been used to examine and describe dynamic patterns of infectious diseases such as influenza and measles (10). 

Since the onset of the COVID-19 pandemic, researchers have applied DMD to examine the spatiotemporal characteristics of the spread of COVID-19 (11,12) and found that changes in the mobility of people over geographic space influence its spread (13). However, these studies focused on either a single US state (Florida) or a single nation (South Korea, a country with a land area smaller than Florida). These studies were not designed to show patterns of large-scale migration of COVID-19 between larger geographic regions.

We used DMD to investigate the dynamic pattern of COVID-19 in the US. Specifically, DMD decomposes the spatiotemporal evolution of the number of COVID-19 cases into linear combinations of simple spatial modes that reveal hidden dynamic behaviors. Each mode represents a distinct basis vector, and each element in the vector indicates the contribution of the corresponding state to the number of COVID-19 cases associated with that mode. Multiplying each mode vector by eigenvalues and a time-dependent coefficient vector and then summing them all will produce the number of COVID-19 cases for each state at different times. Our study aimed to use the features of the extracted modes to describe patterns of the number of COVID-19 cases. Knowledge about these early-stage patterns can inform public health officials and policy makers intervening on COVID-19 and future pandemics to help mitigate transmission across populations.

## Methods

We first collected data on the number of COVID-19 cases in the US from April 6, 2020, to October 9, 2020 (187 days), from the COVID-19 Tracking Project (14) and normalized them to present a clear view for comparison between different geographic areas by using the following equation:

X* = (*X* – min(*X*))/(max(*X*) – min(*X*))

where *X* is a matrix of the cumulative number of COVID-19 cases, with rows for states and columns for days, and X* is the normalized case number. The selection of the cumulative number of COVID-19 cases is to ensure a stable and accurate representation of disease spread by DMD modes (spatial patterns or structures). An alternative would have been to analyze daily incidence data, but these data fluctuate strongly and are difficult to model through DMD. Next, we applied the theory of DMD on infectious diseases (10) to conduct the DMD analysis on COVID-19 data and took the following steps:

Step 1: Create matrices *X*_1_ and *X*_2_ with 1 shifted time step based on the data on number of COVID-19 cases (ie, *X*_1_ = [*x*_1_, *x*_2_, . . ., *x*_n −1_], *X*_2_ = [*x*_2_, *x*_3_, . . ., *x*_n_]) where *x*_i_ is a column vector in a time sequence at time step *i*, with each element representing the count of COVID-19 cases at a specific geographic location.

Step 2: Conduct singular value decomposition, a matrix factorization technique that expresses a matrix as a combination of singular vectors and singular values on the matrix *X*_1_, and use the results to build the approximation A matrix such that *X*_2_ ≈ *AX*_1_.

Step 3: Decompose the approximation A matrix into eigenvectors and eigenvalues; then obtain DMD modes. Eigenvectors are special vectors that change in magnitude only when multiplied by a matrix, and eigenvalues are the corresponding scaling factors for those eigenvectors.

Step 4: Analyze the properties of the DMD modes to investigate the spatiotemporal dynamics of the cumulative number of COVID-19 cases. Data on the cumulative number of COVID-19 cases can be reconstructed as a linear combination of the product of DMD modes, eigenvalues, and time-dependent–mode amplitudes and coefficients.

The distributions of eigenvalues on the eigenvalue spectrum demonstrate their spatiotemporal behaviors, such as the increase, decrease, and periodic fluctuations or variations in disease incidence or prevalence over time. In each dynamic mode vector, every element has 2 critical components: magnitude and angle. The magnitude of each element in the mode vector associated with each state indicates the degree of contribution of that mode to the total cumulative number of COVID-19 cases in that state. The larger the magnitude for that mode, the more contribution the mode makes to the total number of COVID-19 cases for that state. The phase of the element in the mode vector (ie, the angle between the real and imaginary components of the element) indicates the phase difference between that state relative to other states oscillating at the frequency associated with that mode. The smaller the phase difference between 2 states, the closer they oscillate together (10).

The proper modes were chosen based on the value of $\lambda_{k}^{\alpha }∥\phi_{k}∥$ (the modes with the largest $\lambda_{k}^{\alpha }∥\phi_{k}∥$) where α is set as 20, representing a scaling factor to avoid those rapid decaying modes on the system. Further details can be seen in previous studies (6,10). The spatial resolution was determined by state, and the temporal resolution was defined by days. The plots of the eigenvalue spectrum were in a complex plane where *x* and *y* coordinates corresponded to the real and imaginary parts of the eigenvalue, respectively. 

We used MATLAB version 2020 (MathWorks) codes to plot the total number of COVID-19 cases and daily increments (the daily increase in number of COVID-19 cases) in the 50 US states, the District of Columbia, and 5 US territories (American Samoa, Guam, Northern Mariana Islands, Puerto Rico, US Virgin Islands) from April 6, 2020, to October 9, 2020. For simplicity, this article refers to all 56 jurisdictions as states. We also created separate plots for 6 states: California, Florida, Georgia, North Dakota, South Dakota, and Texas. We selected these 6 states because they demonstrated distinct patterns of spread in COVID-19 cases during the study period, with California, Florida, Georgia, and Texas standing out due to their large populations and unique spikes in cases and North Dakota and South Dakota highlighting late-period surges. We visualized both the raw and normalized data on COVID-19 cases and developed DMD modes. These visualizations allowed us to depict aggregated data on the number of COVID-19 cases and the dynamic patterns of these cases in the US.

We scripted MATLAB codes to process data and execute DMD analysis, and we used the MATLAB mapping toolbox to visualize the results on the maps.

## Results

**Figure 1:**
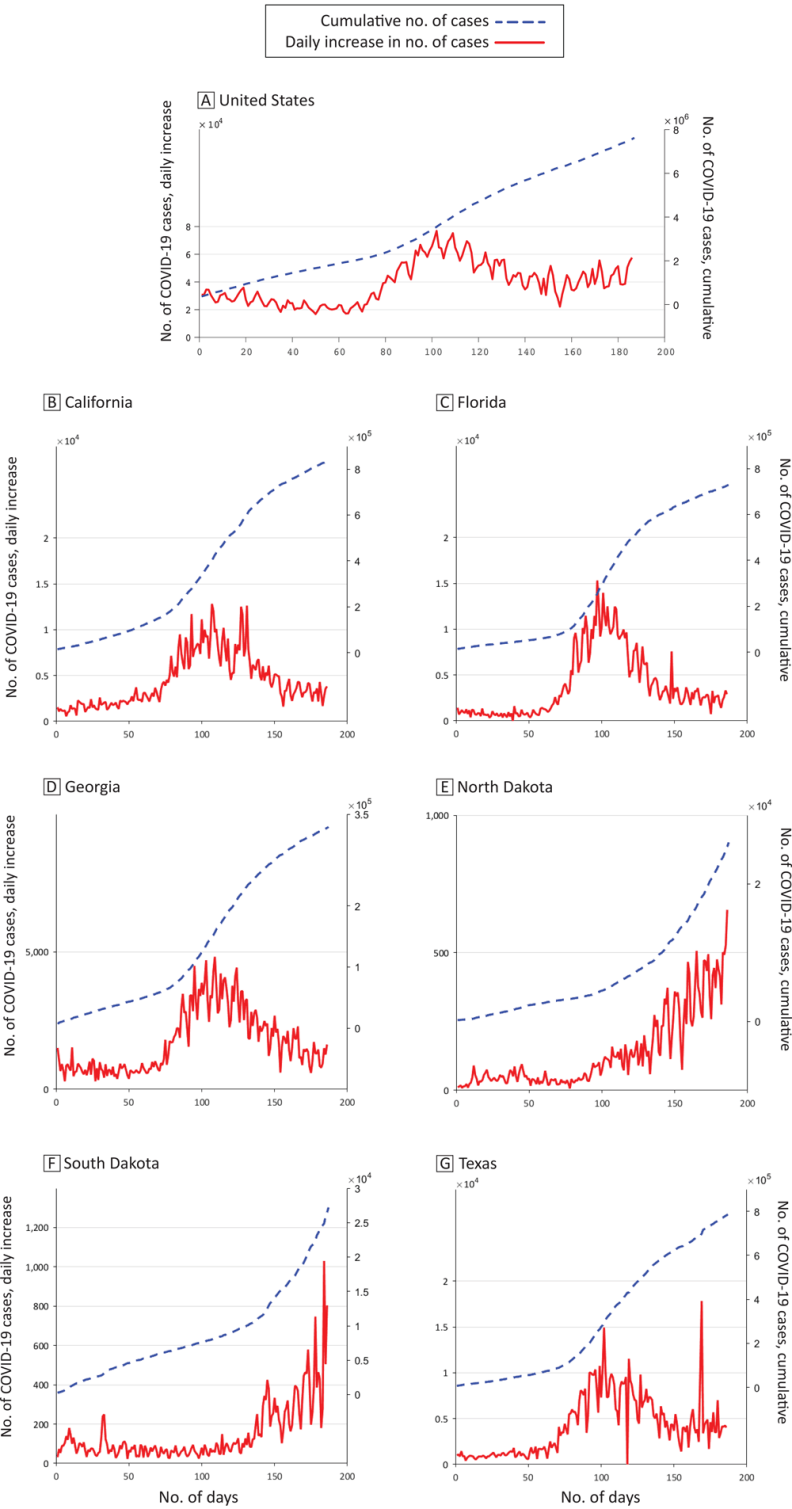
The cumulative number of COVID-19 cases (dashed line) and daily increments (solid line) in the US (A) and in 6 states, April 6, 2020, to October 9, 2020. B, California. C, Florida. D, Georgia. E, North Dakota. F, South Dakota. G, Texas. Data source: COVID-19 Tracking Project (14).

In general, in the 6 states studied, the cumulative number of COVID-19 cases increased slowly in the first 100 days and then increased quickly in the remaining days for nearly all 6 states (Figure 1). California, Florida, Georgia, and Texas showed peaks in daily increments around 100 days, while North Dakota and South Dakota kept increasing during the study period.

**Figure 2:**
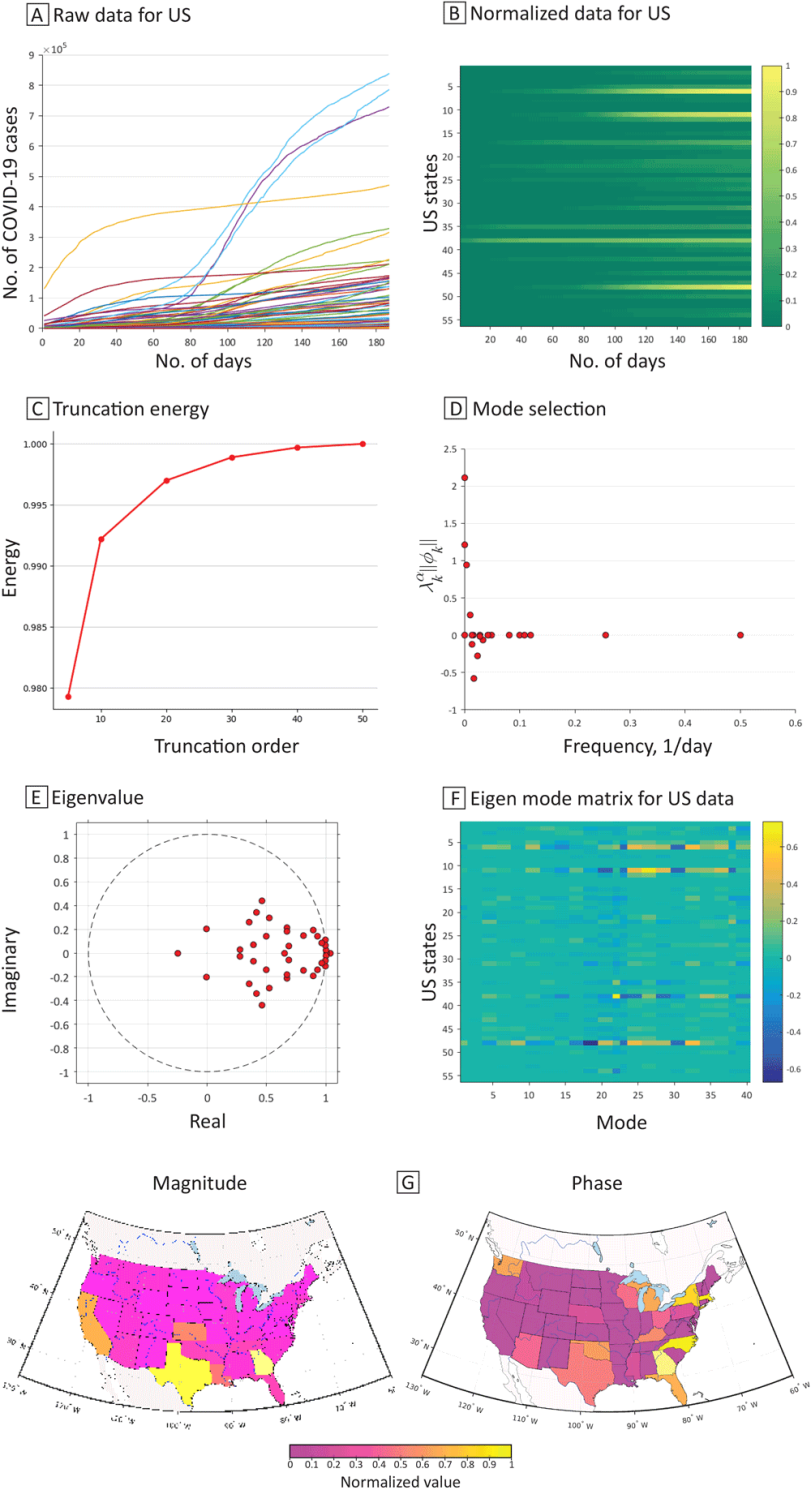
Dynamic mode decomposition analysis of COVID-19 transmission in the US, April 6, 2020, to October 9, 2020. A, The spread of COVID-19 cases in each state, territory, and the District of Columbia. B, The normalized data for each state, territory, and District of Columbia. C, Truncation energy. D, The plot $\lambda_{k}^{\alpha }∥\phi_{k}∥$ versus frequency. E, The eigenvalue spectrum. Dots in the circle indicate decaying of COVID-19 cases, dots on the circle indicate oscillating of COVID-19 cases, and dots outside of the circle indicate spreading of COVID-19 cases. F, The eigen mode matrix for the US data indicates the contribution from each geospatial location. G, The magnitude of the selected mode (the dominant mode with ω = 0, $\lambda_{k}^{\alpha }∥\phi_{k}∥$ = 2.1147) that has the highest $\lambda_{k}^{\alpha }∥\phi_{k}∥$. The magnitude plot shows that California, Louisiana, Kansas, Georgia, and Texas have more COVID-19 cases than other states. Phase plot indicates that Arizona, Florida, Texas, New York, and Washington State were arriving at the peak of COVID-19 cases close in time. Data source: COVID-19 Tracking Project (14).

Figure 2 shows the DMD analysis for COVID-19 data in the US. Specifically, Figure 2A presents the aggregated raw data on the number of COVID-19 cases by state. Figure 2B shows the normalized data, with each row representing a state. Rows are ordered from top to bottom alphabetically by the postal state abbreviation for each state: Alaska (AK) is at the top in the first row, and Wyoming (WY) is at the bottom in the last row. California, Florida, and Texas (corresponding to bright yellow rows) clearly show an increase in COVID-19 cases after approximately 100 days in August 2020. New Jersey and New York State had a relatively high number of COVID-19 cases, which did not increase much during the study period. The plot for truncation energy (Figure 2C) indicates that the selected truncation order (reducing the size of the matrix while still conserving sufficient data for accurate and efficient decomposition) was sufficient for our DMD analysis. Truncation energy is defined as the ratio of cumulative sum of the magnitude of the selected eigenvalues over the sum of all the eigenvalue magnitude. The truncation energy value is 99.97% when the truncation order is set at 40. From the implementation of DMD, we conducted mode selection (Figure 2D) according to their frequencies and the spectrum of the eigenvalues (Figure 2E). The spectrum of the eigenvalues shows that many pairs of eigenvalues are inside the unit circle, and thus have decaying characteristics (ie, a temporal reduction in the number of COVID-19 cases). Some pairs of eigenvalues are on the border; these pairs will neither grow nor decay and will provide oscillatory characteristics if the imaginary part of the eigenvalue is not zero. A few eigenvalues reside outside of the unit value, indicating growing characteristics. Figure 2F shows the visualization of the eigen mode matrix for 56 regions (including states and US territories) with the truncation order set at 40. 

Figure 2G shows a dynamic pattern of COVID-19 case numbers from the dominant mode (ie, frequency = 0), as represented by magnitude and phase of the dominant mode, which is scaled by $\lambda_{k}^{\alpha }∥\phi_{k}∥$. Magnitude is a measure of a state’s contribution to COVID-19 transmission; the map shows a pattern of high magnitude in California, Louisiana, Kansas, Georgia, and Texas. Phase describes the timing and relative position of COVID-19 spread within the region that the mode captures. In the map of phase, regions with similar colors can be viewed as a well-connected group, indicating that these regions simultaneously experienced the spread of COVID-19 (in phase) at the oscillating frequency associated with the mode, even though these regions may not be geographically connected. States that were not neighbors but shared similar phase information, such as Arizona, Florida, Texas, and Washington, or California and Maine, were connected in a way that may have resulted from the transportation of COVID-19 patients or from coincidence.

## Discussion

Our study illustrates the application of DMD in analyzing early data on the COVID-19 pandemic. DMD allowed us to examine the underlying patterns of the spread of COVID-19 without requiring assumptions about the transmission mechanism or prior knowledge of the epidemiology of the disease. As a data-driven tool, DMD is versatile and can accommodate various data formats and units of measurement, such as time series, spatial, and multivariate data, and even irregularly sampled data, as long as the data are consistent in the dataset. As such, DMD is suitable for exploring transmission patterns of epidemiologic diseases. Particularly in the early stages, when a pathogen’s characteristics are not well-defined and the transport of infected, exposed, or asymptotic patients can spread the disease in nonadjacent geolocations, DMD can identify coherent spatiotemporal patterns and dynamic modes that represent dominant behaviors in disease spread for different geographic areas. DMD can also facilitate short-term forecasting of infectious disease dynamics. Such analyses and their results provide public health professionals and policy makers with knowledge to make better-informed decisions about strategies to mitigate disease transmission.

In this study, we used DMD and COVID-19 data to examine dynamic patterns of the spread of COVID-19. The early pandemic strongly affected California, Louisiana, Kansas, Georgia, and Texas, according to the magnitude analysis. The phase map showed the simultaneous increase of COVID-19 cases in Arizona, Florida, Georgia, Massachusetts, New York, North Carolina, Oklahoma, Texas, and other states. This pattern is consistent with the timeline reported on March 3, 2020, by the Centers for Disease Control and Prevention: 60 cases of COVID-19 across Arizona, California, Florida, Georgia, Illinois, Massachusetts, New Hampshire, New York, Oregon, Rhode Island, Washington, and Wisconsin (15). Our results demonstrated patterns of early COVID-19 transmission that were similar to patterns demonstrated by other studies that used different models. For example, McMahon and colleagues (5) applied a spatial correlation analysis on new active cases and found that from April 2020 to October 2020, the epidemic did not progress uniformly: counties in California and Texas had a greater increase than other states in the number of COVID-19 cases. In another study, which used k-means clustering and principal component analysis (16), California and Texas shared similar transmission patterns from March 1 to May 31, 2020, and were grouped into the same cluster. This finding is notable given the close connections of the 2 states and similar containment and mitigation strategies adopted early in the pandemic (17).

### Limitations

Our study has several limitations. One limitation of DMD is its fundamentals of linearity; the data are analyzed on the basis of an approximation of linear relationships, which may only sometimes be the case in real-world applications. For example, the per-capita analysis and the daily incidence analysis did not show accurate reconstructed results by DMD modes. A possible reason for the unreliable results obtained by the per-capita analysis could be population differences. States vary substantially in population size. In addition, populations are not homogeneous in demographic composition; for example, health care needs differ among age groups because of different health concerns. Additionally, the complex spatial structures of COVID-19 transmission patterns, such as the emergence of new variants and the effect of local medical resources and responses, may challenge the ability of DMD to accurately model the spreading of COVID-19 in long-term surveillance of pandemics. Future studies can include various types of data, such as data on use of health care resources, the number of COVID-19 test kits allocated, the number of vaccines administered, and use of personal protective equipment. Such enhanced data could help the DMD model produce more detailed insights into the pandemic’s characteristics, all of which could aid decision makers in developing more effective responses.

### Conclusion

Our study provides insights into the transmission dynamics of COVID-19 in the US and can inform the development of evidence-based public health policies and interventions for COVID-19. Our findings can contribute to a better understanding of the characteristics of early COVID-19 transmission and provide decision makers with timely and accurate information to respond to the pandemic and future public health emergencies.
